# Indents

**Published:** 1911-05-15

**Authors:** 


### INDENTS.

☞ **Brief Articles of Technical or Scientific Value will receive notice and comment. Contributions invited.**

*Aseptic Root Filling.* I made some experiments to ascertain whether I could fill root canals aseptically. There were only two ways in which I could attain aseptic conditions in root canals, and that was first, by treating root canals for three days in succession with tricresol, every time placing a rubber dam in position, and then the tooth was washed with alcohol, bi-chlorid and formalin in order that I might get the external portion of the canal in an aseptic condition. The tri-cresol was placed in and sealed up with cement. The other method is to take the Cochran preparation of gutta-percha, gutta-percha dissolved in chloroform. The chloroform is allowed to evaporate from the mass. The mass of gutta-percha is then mashed and worked with a pestle, and added to that is either the eucalyptol or cajuput, or some of the essential oils. I tried all the balsams, but balsam of Peru is the only one that retains for an indefinite period a disinfectant property. The canals are left practically hermetically sealed with gutta-percha and balsam of Peru. After the preparation is made the bottles are kept perfectly stopped. I found this to be antiseptic by taking platinum broaches and sterilizing the broach by passing it through a Bunsen burner, and inoculating a culture tooth from that. In a few teeth that were to be extracted I did fill the root canals without any special preparation, and disinfected them, and with the exception of the cases where I used balsam of Peru, the canals were not aseptic. The method of using gutta-percha and balsam of Peru is this: Anything that volatilizes at all will shrink. This preparation of gutta-percha evaporates less than any I have seen. I take the balsam of Peru on a glass slab, work it in the canals, and follow that with a gutta-percha preparation, and follow that with cones or points. There is this difficulty, that it is irritating, if it volatilizes; however the soreness that follows the fillings of the root may not appear for two or three weeks after the root is filled. In a few instances I have known the tooth to get very sore. After the uneasiness disappears then the tooth is at perfect rest. I have been using the preparation about five years. When Dr. Bentley got to discussing whether or not you could render aseptic the conditions I know you cannot by Dr. Buckley's method because, as Dr. Bentley said, there is nothing in this room or any other room unless it is prepared every day, that is in an aseptic condition. The object of every precaution is to minimize the number of bacteria. There are always two penalties in filling a root canal that you may be certain of. It if makes any trouble you may be certain that you have not disinfected it. The filling of root canals is a good deal of nonsense, in a way. No small root canal I have ever seen, that has been sealed properly makes trouble. It is either left infected, or is re-infected, if it makes troubles. In the small canals I do not believe there is any liability unless pressure forces the pulp through. As long as there is pressure behind, which must necessarily be, the serum will percolate through and into these canals. The main object in filling canals is to lessen space. It should be disinfected absolutely before it is filled. Balsam of Peru was introduced by a German some years ago. I do not make any claims for it. I have given Dr. Dittmar some of the preparation and asked him to use it. I have used it several years, and I have the first case to find which I rendered aseptic reinfected.—Dr. G. W. Cook, in *Review*.

*Root Impression.* In taking the impression of a root Dr. Van Woert uses a copper band somewhat larger than the root. It is trimmed to follow the line of the gum. By forcing impression compound into it and against the root a direct pressure is obtained on the impression material which insures an accuracy that nothing else will.—*Review*.

